# Antibacterial and Antifungal Activities of Linear and Cyclic Peptides Containing Arginine, Tryptophan, and Diphenylalanine

**DOI:** 10.3390/antibiotics14010082

**Published:** 2025-01-13

**Authors:** David Salehi, Eman H. M. Mohammed, Naiera M. Helmy, Keykavous Parang

**Affiliations:** 1 Center for Targeted Drug Delivery, Department of Biomedical and Pharmaceutical Sciences, Chapman University School of Pharmacy, Harry and Diane Rinker Health Science Campus, Irvine, CA 92618, USA; dsalehi@chapman.edu (D.S.); emohammed@chapman.edu (E.H.M.M.); nm.helmi@nrc.sci.eg (N.M.H.); 2 Chemistry Department, Faculty of Science, Menoufia University, Shebin El-Koam 51132, Egypt; 3 Microbial Biotechnology Department, Biotechnology Research Institute, National Research Centre, Giza 12622, Egypt

**Keywords:** antibiotic resistance, antibiotics, antifungal, antimicrobial peptides, arginine, 3,3-Diphenyl-L-alanine, tryptophan, unnatural amino acids

## Abstract

**Background.** We have previously reported peptides composed of sequential arginine (R) residues paired with tryptophan (W) or 3,3-diphenyl-L-alanine residues (Dip), such as cyclic peptides [R_4_W_4_] and [R_4_(Dip)_3_], as antibacterial agents. **Results.** Herein, we report antibacterial and antifungal activities of five linear peptides, namely ((DipR)_4_(WR)), ((DipR)_3_(WR)_2_), ((DipR)_2_(WR)_3_), ((DipR)(WR)_4_), and (DipR)_4_R, and five cyclic peptides [(DipR)_4_(WR)], [(DipR)_3_(WR)_2_], [(DipR)_2_(WR)_3_], [(DipR)(WR)_4_], and [DipR]_5_, containing alternate positively charged R and hydrophobic W and Dip residues against fungal, Gram-positive, and Gram-negative bacterial pathogens. The minimum inhibitory concentrations (MICs) of all peptides were determined by the micro-broth dilution method against *Methicillin-Resistant Staphylococcus aureus*, *Klebsiella pneumoniae*, *Pseudomonas aeruginosa*, *Escherichia coli*, *Staphylococcus aureus*, *Enterococcus faecium*, *Enterococcus faecalis*, *Streptococcus pneumoniae*, and *Bacillus subtilis*. Fungal organisms were *Candida albicans*, *Candida parapsilosis*, and *Aspergillus fumigatus.* [DipR]_5_ and ((DipR)_2_(WR)_3_) showed MIC values of 0.39–25 µM and 0.78–12.5 µM against Gram-positive and Gram-negative bacteria strains, respectively. The highest activity was observed against *S. pneumoniae* with MIC values of 0.39–0.78 µM among tested compounds. [DipR]_5_ demonstrated MIC values of 6.6 µM against *C. parapsilosis* and 1.6 µM against *A. fumigatus*, whereas fluconazole showed MIC values of 3.3 µM and >209 µM, respectively. **Conclusions.** These findings highlight the potential of these peptides as broad-spectrum antimicrobial agents.

## 1. Introduction

The prevalence of bacterial resistance has become one of the most serious public health crises because of the limited availability of effective antibiotics to treat them. The rise and spread of antibiotic-resistant infections in hospitals and communities have challenged the effectiveness of current antibiotic stewardship strategists to face this obstacle [1,2,3].

The Center for Disease Control has reported that more than 2.8 million antibiotic-resistant infections occur in the U.S. each year, with a mortality rate of more than 35,000 people [4]. Multidrug-resistant bacteria are responsible for roughly half of the 37,000 deaths a year in the 27 member states of the European Union that are caused by infections associated with hospital care [5]. The study only focuses on infections related to hospital care and does not count community-acquired infections. Pathogens causing hospital-acquired infections often display higher levels of antibiotic resistance compared to those responsible for community-acquired infections. This is largely due to prolonged antibiotic exposure and selective pressure in hospitals [6]. Thus, these data show that the discovery of novel treatment alternatives to commercially available antibiotics is urgently required to tackle the challenges of bacterial resistance.

Methicillin-Resistant *Staphylococcus aureus* (MRSA) is associated with severe infections [7,8]. *Klebsiella pneumoniae* (KPC) causes conditions such as pneumonia, bloodstream infections, and meningitis and has developed resistance to carbapenems, a last-line class of antibiotics [9]. Multidrug-resistant *Pseudomonas aeruginosa* (PSA) caused approximately 32,600 infections and 2700 deaths in the United States in 2017, primarily affecting hospitalized patients [10]. *Escherichia coli* (*E. coli*) is responsible for diarrhea, urinary tract infections, and other illnesses with frequent outbreaks linked to contaminated or undercooked foods [11]. *Enterococcus faecalis* (*E. faecalis*) can withstand harsh environmental conditions and cause endocarditis and urinary tract infections [12,13,14]. *Streptococcus pneumoniae* (*S. pneumoniae*), despite available vaccines, remains a leading cause of otitis media, pneumonia, meningitis, and community-acquired bacteremia, particularly in children and older adults [15].

Antimicrobial peptides (AMPs) are part of the defense system of the infected organism. AMPs are considered promising candidates to fight multidrug-resistant bacterial pathogens due to their excellent broad-spectrum antibacterial activity and non-specific bacterial membrane rupture mechanism, which may not allow bacterial or fungal pathogens to develop resistance [16,17,18,19]. The endogenous AMPs of plants and animals are typically cationic (i.e., contain excess lysine and arginine residues) and amphipathic molecules [20]. Two major classes of peptides in human skin are reported β-defensins and cathelicidins, which have antimicrobial activity against bacterial, fungal, and viral pathogens. These peptides, produced by keratinocytes in the skin cells, disrupt the membrane of the target microbe and penetrate the microbial membrane [21]. This unspecific mode of action is suggested to be responsible for the broad-spectrum activity of many antimicrobial peptides [22]. *Saccharum officinarum* is a plant that produces an AMP called sugarcane defensin 5, which is associated with antifungal property [23].

Mechanistically, AMPs act primarily by increasing membrane permeability and forming pores, which lead to microbial cell death. Their cationic and amphiphilic nature also align them closely with cell-penetrating peptides (CPPs), which are used for intracellular delivery [24,25,26]. CPPs maintain the formed pores in the plasma membrane open for a shorter period of time compared to AMPs. Therefore, delivery of the intended reagents could be accomplished with the least toxicity to the cells in the case of CPPs.

This work builds on our previously published study on cell-penetrating peptides composed of arginine, tryptophan, and diphenylalanine (Dip). In our previous work, we explored in vitro cytotoxicity, cell-penetrating capabilities, uptake mechanisms, secondary structures, nanostructure formation, and molecular transporter properties [26]. Building on this foundation, the current study specifically focuses on evaluating their antibacterial and antifungal activities. The rationale for this investigation stems from the peptides’ amphiphilic properties, which are not only integral to their cell-penetrating abilities, but also play a key role in their antimicrobial efficacy against both bacterial and fungal pathogens.

We have previously reported the synthesis of [E_4_W_4_], [KR_5_], [F_4_R_5_], [Y_4_R_4_], and [R_4_W_4_] and tested them against bacterial pathogens. Among all the synthesized peptides, [R_4_W_4_] containing arginine and tryptophan residues in a sequential manner showed the most potent antibacterial activity against MRSA, exhibiting a minimal inhibitory concentration (MIC) of 2.67 μg/mL [27,28]. [R_4_W_4_] also exhibited antibacterial activity against a broad number of bacteria, including both Gram-negative and Gram-positive bacteria [24,27,28,29,30,31]. [R_4_W_4_] demonstrated bactericidal activity and showed a synergistic effect with gentamicin against *E. coli*. The mechanism of action against MRSA involved changes in zeta potential, membrane depolarization, and binding to lipoteichoic acid (LTA), with concentration-dependent membrane perturbations [31]. Similarly, Mohammed et al. (2022) introduced a potential strategy for treating multidrug-resistant pathogens by combining [W_4_KR_5_] and a variety of classical antibiotics to improve the antibacterial effectiveness [24]. These findings suggest that cyclic peptides with sequential tryptophan and arginine residues are promising frameworks for designing effective AMPs and antibacterial agents.

Our laboratory has also reported the antibacterial activities of peptides containing sequential arginine and hydrophobic residues, including tryptophan, 3,3-diphenyl-L-alanine (Dip), 4,4′-biphenyl-Lalanine (Bip), 3-(2-naphthyl)-L-alanine (Nal), as well as peptide–antibiotic conjugates [28,32,33]. For instance, the bicyclic peptide [W(WR)_4_K]-[W(WR)_4_E] demonstrated activity against Gram-positive bacteria, including MRSA (ATCC BAA-1556) and *S. aureus* (ATCC 29213) with MIC values of 4.8–9.6 µM [34].

The primary objective of this study is to evaluate these peptides as broad-spectrum antimicrobial agents targeting both bacterial and fungal pathogens. The dual activity stems from their amphiphilic nature and cationic charge, which enable interactions with the negatively charged microbial membranes. These properties allow the peptides to disrupt membrane integrity, leading to cell lysis in both bacterial and fungal cells [16,35,36].

The differences in activity between antibacterial and antifungal properties may arise from the structural and compositional differences in bacterial and fungal membranes. Bacterial membranes are rich in phospholipids [37], such as phosphatidylglycerol and cardiolipin, whereas fungal membranes contain ergosterol [38] and other sterol components that influence membrane fluidity and integrity. These variations in membrane composition could explain why certain peptides show varying levels of potency against bacterial versus fungal pathogens.

**Figure 1:**
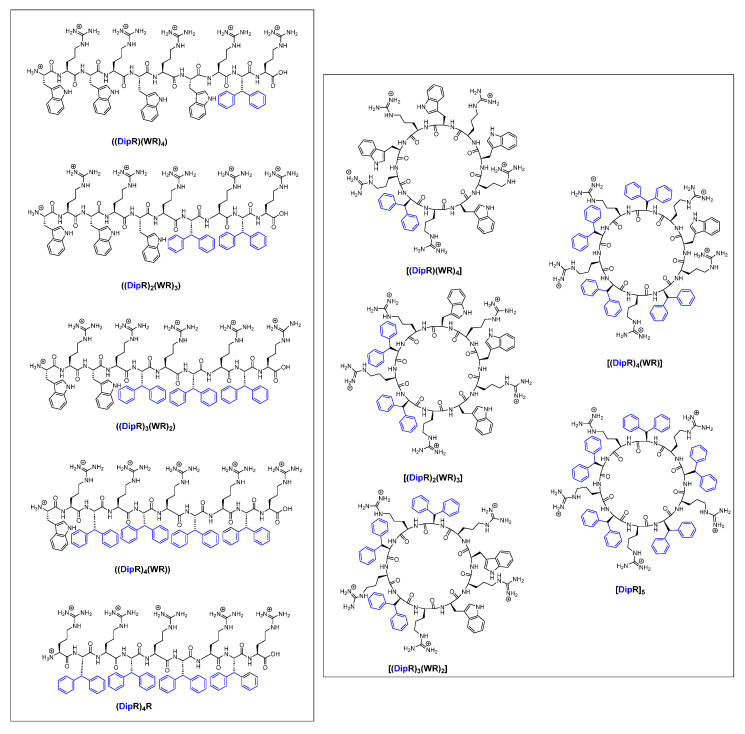
Chemical structures of synthesized cyclic and linear peptides containing R, Dip and/or W residues (Parentheses () and brackets [] represent the linear and cyclic peptides, respectively). Adapted from [26]. Hydrophobic side chains of Dip are shown in blue.

In continuation of our efforts to design novel AMPs with improved properties, we report here the evaluation of the linear and cyclic peptides containing alternative positively charged arginine residues and hydrophobic residues (W and Dip) (Figure 1) against pathogenic bacteria and fungi (Table 1 and Table 2). Herein, we report the activities of the linear peptides ((DipR)_4_(WR)), ((DipR)_3_(WR)_2_), ((DipR)_2_(WR)_3_), ((DipR)(WR)_4_), and (DipR)_4_R, as well as that of the cyclic peptides [(DipR)_4_(WR)], [(DipR)_3_(WR)_2_], [(DipR)_2_(WR)_3_], [(DipR)(WR)_4_], and [DipR]_5_ to determine the effect of replacing of W with Dip in antimicrobial and antifungal properties.

We hypothesized that these peptides can be used as a broad-spectrum antibiotic and antifungal agents by interacting with bacterial and fungal membranes. The head-to-tail cyclization and incorporation of unnatural amino acids could provide improved stability against degradation in the biological environment. Cyclic peptides are generally more stable than their linear counterparts due to their resistance to proteolytic degradation. For example, we have previously shown the plasma stability of [DipR]_5_. Approximately 15% degradation of [DipR]_5_ was observed within the first hour, but the rate of degradation significantly slowed by the two-hour mark. These results highlight the peptide’s high stability, likely attributed to its cyclic structure and the incorporation of unnatural amino acids, which enhance resistance to enzymatic breakdown [26].

The peptides were evaluated against MRSA (ATCC BAA-1556) and some of the ESKAPE pathogens, including *Enterococcus faecium* (*E. faecium*) (ATCC 700221), *Staphylococcus aureus* (*S. aureus*) (ATCC 29213), *K. pneumoniae* (ATCC BAA-1705), *P. aeruginosa* (ATCC 27883), and some other pathogenic bacteria, such as *E. coli* (ATCC 25922), *E. faecalis* (ATCC 29212), *S. pneumoniae* (ATCC 51938), and *Bacillus subtilis* (*B. subtilis*) (ATCC-6633). The peptides were also evaluated against pathogenic fungi, e.g., *Candida parapsilosis* (*C. parapsilosis*) (ATCC 22019), *Aspergillus fumigatus* (*A. fumigatus*) (Af-293), and *Candida albicans* (*C. albicans*) (ATCC 60193), to determine the broad-spectrum antifungal activities of the compounds.

## 2. Results and Discussion

### 2.1. Antibacterial Activity

The antibacterial activity of all ten synthesized linear and cyclic peptides was evaluated against MRSA (ATCC BAA-1556), *S. aureus* (ATCC 29213), *E. faecium* (ATCC 700221), *S. pneumoniae* (ATCC 51938), *E. faecalis* (ATCC 29212), *B. subtilis* (ATCC-6633), *K. pneumoniae* (ATCC BAA-1705), *P. aeruginosa* (ATCC 27883), and *E. coli* (ATCC 25922). Meropenem and daptomycin were used as positive controls for this study. Certain peptides are marked as “NT” (not tested) against specific bacterial strains based on initial screening data. Peptides that demonstrated relatively lower antibacterial activity or a narrower spectrum in preliminary experiments were not prioritized for further testing against all bacterial strains due to resource constraints and to focus on the most promising candidates. The MIC was determined by micro-broth dilution protocol, where the minimal concentrations were determined to be in wells with no visible bacterial growth.

Among the tested peptides, [DipR]_5_ showed promising MIC values of 0.39–6.25 µM (0.74–11.9 µg/mL) against Gram-positive bacteria strains: MRSA, *S. aureus*, *E. faecium*, *E. faecalis*, *S. pneumoniae*, and *B. subtilis*. [DipR]_5_ showed moderate MIC values of 12.5–25 µM (23.8–47.6 µg/mL) against Gram-negative strains: *K. pneumoniae*, *P. aeruginosa*, and *E. coli* (Table 1).

The observed differences in activity of [DipR]_5_ against Gram-positive versus Gram-negative bacteria are likely due to the fundamental structural differences between their phospholipid composition in their membranes [39]. These structural differences explain why [DipR]_5_ showed significantly higher potency against Gram-positive bacteria compared to Gram-negative strains. Gram-positive and Gram-negative bacteria exhibit distinct differences in their phospholipid composition, which influence their interactions with AMPs. Gram-positive bacteria primarily contain phosphatidylglycerol (PG), cardiolipin (CL), and lysyl-phosphatidylglycerol (LPG) as dominant phospholipids. These molecules contribute to the negatively charged cell membrane, which facilitates electrostatic interactions with AMPs, such as [DipR]_5_. In contrast, Gram-negative bacteria feature phosphatidylethanolamine (PE) as the predominant phospholipid, along with PG and CL, and an additional outer membrane containing lipopolysaccharides (LPS) [40]. This outer membrane acts as a protective barrier, reducing AMP access to the inner membrane.

Cyclic peptide [DipR]_5_ was found to be active against the bacteria in the order of *S. pneumoniae* (MIC = 0.39 µM) *˃ E. faecalis* and *E. faecium* (MIC = 0.78 µM) *>* MRSA and *S. aureus* (MIC = 3.1 µM) > *B. subtilis* (MIC = 6.3 µM) > *E. coli* (MIC = 12.5 µM) > *K. pneumoniae*, and *P. aeruginosa* (MIC = 25 µM).

The linear peptides, on the other hand, showed different levels of activity, with some peptides, such as (DipR)_4_R, demonstrating potent effects against certain bacterial strains such as *MRSA* and *K. pneumoniae*. The corresponding linear peptide (DipR)_4_R showed activity in the order of *S. pneumoniae* (MIC = 0.39 µM) *˃* MRSA, *S. aureus*, *E. faecium*, and *Bacillus subtilis* (MIC = 1.6 µM) *˃ E. faecalis* (MIC = 3.1 µM) > *K. pneumoniae* and *E. coli* (MIC = 6.3 µM) > *P. aeruginosa* (MIC = 12.5 µM). These data indicate that the linear peptide (DipR)_4_R was more potent against a number of bacterial strains, such as MRSA, *K. pneumoniae*, *P. aeruginosa*, *E. Coli*, *S. aureus*, and *B. Subtilis*, when compared with [DipR]_5_. Indeed, (DipR)_4_R was the most potent peptide among all the compounds against MRSA, *K. pneumoniae*, *E. Coli*, and *S. aureus.* At the same time, [DipR]_5_ was the most potent peptide against *E. Faecium*, *E. faecalis*, and *S. pneumoniae*. Of note, we have previously reported that (DipR)_4_R exhibited more cytotoxicity than other peptides in HEK-293 cells after 24 h incubation, especially above 10 µM [26].

While other peptides proved to be less potent than [DipR]_5_ or (DipR)_4_R overall, all the peptides were very potent against *S. pneumoniae* (MIC = 0.39–0.78 µM) when compared with other bacteria. Furthermore, these peptides showed modest activity against *E. Faecalis* (MIC = 0.78–12.5 µM) and MRSA (MIC = 1.6–6.3 µM). The superior activity of peptides against *S. pneumoniae* compared to other tested organisms can be attributed to several factors, such as bacterial membrane phospholipid composition and stronger peptide-cell membrane interactions. *S. pneumoniae*, a Gram-positive bacterium, has a distinct phospholipid composition, predominantly comprising phosphatidylglycerol (PG) (60%) and cardiolipin (CL) (40%). This differs from other Gram-positive bacteria, such as *E. faecalis*, which has a phospholipid composition of PG (30%), CL (20%), and other components (50%) [39]. These differences in phospholipid profiles enhance the interaction of positively charged peptides, such as [DipR]_5_, with the bacterial cell membrane, facilitating effective membrane disruption [41]. The amphiphilic nature of these peptides enables them to target bacterial membranes efficiently, and the composition of *S. pneumoniae*’s membrane may render it particularly susceptible to such disruption, resulting in enhanced peptide activity. Unlike Gram-negative bacteria, which possess an outer membrane and efflux pumps as defensive barriers, *S. pneumoniae* lacks these features, further contributing to the observed higher potency of the peptide. There are other possibilities for the superior activity of peptides against *S. pneumoniae* that warrant further investigation. One potential factor is the higher membrane potential in *S. pneumoniae*, which could increase the attraction of positively charged peptides like [DipR]_5_, thereby enhancing membrane disruption. Additionally, differences in protease activity among bacteria may play a role; *S. pneumoniae* may lack certain proteases that degrade certain AMPs, making it more susceptible compared to other organisms.

Linear peptide ((DipR)_2_(WR)_3_) was found to show moderate activity against the bacteria in the order of *S. pneumoniae* (MIC = 0.78 µM) *˃ B. subtilis* (MIC = 1.6 µM) > *MRSA*, *S. aureus*, *E. faecium* (MIC = 3.1 µM) > *E. faecalis* and *E. coli* (MIC = 6.3 µM) > *K. pneumoniae* and *P. aeruginosa* (MIC = 12.5 µM). These findings further highlight the variability in peptide efficacy across bacterial species and the potential impact of structural features on activity.

The peptides demonstrated varying degrees of potency and displayed comparable or superior activity to meropenem against *K. pneumoniae*, *E. faecium*. Most peptides were more potent than daptomycin against *K. pneumoniae*, *P. aeruginosa*, *E. coli*, *E. faecalis*, and *S. pneumoniae*. Furthermore, [DipR]_5_ was more potent than daptomycin against *E. faecium*, *E. faecalis*, and *S. pneumoniae*, suggesting that the peptide could serve as a potential alternative or complementary agent in treating resistant bacterial infections. Overall, these compounds can be regarded as strong candidate compounds against *S. pneumoniae* and MRSA, with MIC values of 0.39–0.78 µM and 1.6–6.3 µM, respectively, for further optimization.

The mechanism of action of the peptides was inferred based on their amphiphilic and cationic properties, which are characteristic of many AMPs, as discussed in the introduction. The positively charged arginine residues facilitate electrostatic interactions with the negatively charged bacterial and fungal membranes, allowing the peptides to bind to the microbial surface. Subsequently, the hydrophobic residues (tryptophan and diphenylalanine) promote insertion into the lipid bilayer, disrupting membrane integrity. This process likely results in pore formation, increased membrane permeability, and eventual cell lysis. As demonstrated for [R_4_W_4_], the mechanism of action of these compounds against bacteria is expected to involve changes in zeta potential, membrane depolarization, and concentration-dependent membrane perturbations [31]. The slightly lower activity against some strains may be due to differences in peptide binding affinity and/or penetration mechanisms in membranes. Table 1 shows the MIC (µM) of peptides against different bacterial strains (Table 1).

### 2.2. Antifungal Activity

The antifungal activity of all ten synthesized linear and cyclic peptides was evaluated against *C. albicans* (ATCC 60193), *C. parapsilosis* (ATCC 22019), and *A. fumigatus* (AF-293). Fluconazole and amphotericin B were used as positive controls for this study. The MIC was determined at concentrations in wells with no visible fungal growth.

Among the peptides, cyclic peptide [DipR]_5_ exhibited the most potent antifungal activity with MIC values of 1.6–6.6 µM (3.0–12.5 µg/mL) against *C. parapsilosis* and *A. fumigatus* (Table 2). [DipR]_5_ was found to be active against the fungi in the order of *A. fumigatus* (MIC = 1.6 µM) ˃ *C. parapsilosis* (MIC = 6.6 µM) > *C. albicans* (MIC = 13.1 µM). Similarly, [(DipR)_4_(WR)] also showed activity in the order of *A. fumigatus* (MIC = 1.7 µM) ˃ *C. albicans* and *C. parapsilosis* (MIC = 6.7 µM).

Among the linear peptides, ((DipR)_4_(WR)) was notably active against the fungi in the order of *A. fumigatus* (MIC = 1.6 µM) ˃ *C. albicans* and *C. parapsilosis* (MIC = 6.6 µM). Overall, all peptides exhibited the highest potency against *A. fumigatus* (MIC = 1.6–3.5 µM) when compared to their activity against other fungi, which ranged from 3.4 to 28.2 µM. Furthermore, these peptides showed modest activity against *C. parapsilosis* (MIC = 6.6–14.1 µM) and *C. albicans* (MIC = 3.4–28.2 µM).

Notably, fluconazole exhibited no activity against *A. fumigatus* at the highest tested concentration, with an MIC greater than 209 µM, whereas the peptides demonstrated superior efficacy, suggesting their potential as lead compounds for antifungal therapy. While the antifungal activity of these peptides was generally more potent against *A. fumigatus*, they exhibited varying degrees of activity against the Candida species, indicating broad-spectrum antifungal potential with particular strength against *A. fumigatus*. Their activity against *A. fumigatus*, *C. albicans*, and *C. parapsilosis* suggests potential applications as a lead candidate for further investigation as antifungal agents.

We have previously reported in vitro cytotoxicity of the synthesized peptides in a range of mammalian cell lines, including human ovarian adenocarcinoma cells (SK-OV-3), human peripheral blood lymphoblast cells (CCRF-CEM), healthy human embryonic kidney cells (HEK-293), human mammary adenocarcinoma cells (MDA-MB-468), normal pig kidney epithelial cells (LLC-PK1), and human uterine sarcoma fibroblast cells (MES-SA) [26]. The results revealed that peptides containing Dip residues exhibited higher cytotoxicity, particularly against ovarian cancer cells (SK-OV-3), compared to lymphoblast (CCRF-CEM) and healthy kidney cells (HEK-293). Cyclic [DipR]_5_ showed significant cytotoxic effects at 25 µM, reducing proliferation by up to 84% in CCRF-CEM cells after 72 h, while cytotoxicity was minimal at lower concentrations, such as 10 µM. Similarly, the peptides exhibited noticeable cytotoxicity above 25 µM in HEK-293 cells but are non-toxic at concentrations up to 10 µM. The peptides demonstrated selectivity for SK-OV-3 cells, with lower toxicity toward healthy HEK-293 cells. In-depth studies on [DipR]_5_ showed reduced cytotoxicity against MDA-MB-468 and LLC-PK1 cells but moderate effects on MES-SA cells. Overall, the data highlight the peptides’ selective cytotoxic potential and minimal effects on normal cells at lower concentrations. This provides a relatively acceptable therapeutic window for further development. The results indicate that the peptides could be used as broad-spectrum antifungal and antibacterial agents against pathogenic fungi and bacteria with minimal cytotoxicity. However, further evaluations of the compounds on other normal cell lines and animal studies are critical to fully assess their therapeutic potential.

## 3. Materials and Methods

*C. albicans* (ATCC 60193), *C. parapsilosis* (ATCC 22019), *MRSA* (AATCC BAA-1556), multidrug-resistant strans *K. pneumoniae* (ATCC BAA-1705), *P. aeruginosa* (ATCC 27883), *E. coli* (ATCC 25922), *S. aureus* (ATCC 29213), *E. faecium* (ATCC 700221), *E. faecalis* (ATCC 29212), *S. pneumoniae* (ATCC 51938), and *B. subtilis* (ATCC-6633) were obtained from ATCC (Manassas, VA, USA), and propagated as per the recommendation of American Type Culture Collection (ATCC; USA). *A. fumigatus* Af-293 was obtained from Dr. Eric Pearlman from the University of California, Irvine. RPMI 1640 (Lot # RNBG5842, Sigma Aldrich, Sigma Aldrich, St. Louis, MO, USA) with L-glutamine supplemented with 2% D-Glucose (Lot # 160725, Fisher Scientific, Kansas City, MO, USA) and adjusted pH with HEBES buffer (Ref 15630–080, Gibco ThermoFisher Scientific, Grand Island, NY, USA) were used as media for fungal growth. Yeast Peptone Dextrose (YPD) agar (1% *w*/*v* yeast extract (Pcode 102436548, Sigma Aldrich, St. Louis, MO, USA), 2% *w*/*v* peptone (Pcode 102475393, Sigma Aldrich, St. Louis, MO, USA), 2% *w*/*v* D-Glucose, 1.5% Agar (Pcode 102425215, Sigma Aldrich, St. Louis, MO, USA)), and Media from Hardy Diagnostics (Santa Maria, CA, USA) were used as bacterial growth media. Ultrapure water was from a Milli-Q system (Temecula, CA, USA). Synthesis of all the peptides was conducted according to the previously reported procedure by us [26]. The final peptides were confirmed to have a purity of ≥95%, as determined by analytical HPLC. The molecular weights were verified using mass spectrometry (MS) to ensure the precision and accuracy of the synthesis.

### 3.1. Antibacterial Assays

All bacterial experiments were carried out in a laminar flow hood (Labconco, Kansas City, MO, USA). The bacterial strains were cultured following the guidelines set by the Clinical Laboratory Standards Institute (CLSI). Antibacterial assays were performed using a standard microtiter dilution method as described in previous studies [24]. The minimum inhibitory concentration (MIC) refers to the lowest peptide concentration that inhibits bacterial growth in wells with no visible bacterial growth. MIC was determined by micro-broth dilution. Bacteria were cultured overnight in 6 mL of Luria Broth (LB). A stock solution of the peptide was prepared in water and DMSO, and a test compound solution with a concentration of 256 µg/mL was made in LB. Meropenem, with a solubility of 8 mg/mL, was prepared as a stock solution at 4 mg/500 µL. An aliquot of the overnight bacteria culture was diluted in normal saline (NS, 1 mL) until achieving a 0.5 McFarland turbidity (1.5 × 10^8^ bacterial cell CFU/mL). A 0.5 McFarland compared solution (60 µL) was added to Mueller Hinton Broth (MH) 8940 µL to generate a 1/150 dilution. A 200 µL test compound solution was added to the first well of a 96-well plate, and the remaining wells (2–12) were filled with 100 µL of MH media using a multi-channel pipette from a sterile reservoir. A concentration of 100 μM/mL of the tested peptides was prepared from a stock solution of the samples for testing in Muller−Hinton broth MH media. Peptides stock solution was dissolved in 5% DMSO in water, and the final concentration of DMSO in the first well of assay plate was 2.5% DMSO/H_2_O and diluted across the assay plate.

A 100 µL sample from well 1 was pipetted into well 2, and the solution was mixed thoroughly by pipetting up and down. Next, 100 µL was transferred from well 2 to well 3, followed by thorough mixing. This 2-fold serial dilution process continued through well 11, with no additions made to well 12. The final 100 µL from well 11 was discarded. After completing the serial dilution, each well (1–12) contained 100 µL of solution. The bacterial culture in MH media was vortexed and transferred to a sterile reservoir, from which 100 µL was added to each well using a 12-channel pipette. (Note: The dilutions were further diluted upon adding the bacterial solution; for example, the concentration in well 1 started at 512 µg/mL but became 256 µg/mL after the addition of the bacterial culture.) The plates were incubated overnight at 37 °C for 24 h. All experiments were performed in triplicate and repeated twice, with controls consisting of bacteria in MH media and positive controls of daptomycin or meropenem.

### 3.2. Antifungal Assays

*A. fumigatus* (Af-293) was cultured on Sabouraud dextrose agar (SDA) (Lot #3431660, Oxoid) for 48–72 h at 35 °C to obtain fresh and mature colonies which reached the proper sporulation then used to prepare the inoculum. The colonies were covered with Phosphate-Buffered Saline (PBS) containing 0.025% Tween-20, scraped from the plate, and carefully collected into a sterile tube. The suspension was centrifuged at 3000 RPM for 5 min after the separation between conidia and hyphae. The supernatant was discarded, and the pellet was resuspended in 5 mL of PBS.

Peptide concentrations of 50, 25, 12.5, 6.25, and 3.1 μg/mL were tested in triplicate. Each well of a 96-well plate was inoculated with the prepared inoculum suspension, which was diluted with RPMI 1640 medium containing L-glutamine, supplemented with 2% D-glucose, and the pH adjusted using HEPES buffer. The plates were incubated for 50 min at 35 °C to achieve a final cell concentration of 10^6^ cells/mL. The control wells contained the same inoculum without any compound. The plates were then incubated for 24 and 48 h at 35 °C. Fungal growth was visualized, and the minimum inhibitory concentration (MIC) was determined as the lowest peptide concentration, where no growth was observed.

In this study, *C. albicans* and *C. parapsilosis* were cultured on Yeast Peptone Dextrose (YPD) agar containing 1% *w*/*v* yeast extract, 2% *w*/*v* peptone, 2% *w*/*v* D-glucose, and 1.5% agar. A single colony from the agar was selected and cultured in Tryptone soy broth supplemented with 0.1% D-glucose, then incubated for 24 h at 37 °C. The cells were washed twice with PBS by centrifugation at 2000 RPM for 5 min. The pellet was resuspended in 5 mL of PBS, diluted in RPMI 1640 with L-glutamine, supplemented with 2% D-glucose, and the pH adjusted using HEPES buffer. The suspension was then incubated for 50 min at 35 °C to achieve a final cell concentration of 10^6^ cells/mL.

Peptide concentrations of 50, 25, 12.5, 6.25, and 3.1 μg/mL were tested in triplicate and repeated twice. Each well of a 96-well plate was inoculated with the prepared inoculum suspension, which was diluted with RPMI media to a final volume of 200 μL per well. The control well received the same inoculum without any compound. The plates were incubated for 24 h at 37 °C. Fungal and microbial growth were visually observed in the control wells, and the minimum inhibitory concentration (MIC) was determined as the lowest concentration at which no growth was detected in the wells. Amphotericin B was used as a standard antifungal drug.

## 4. Conclusions

The synthesized linear and cyclic peptides demonstrated potent antibacterial and antifungal activities, particularly against *S. pneumoniae*, *MRSA*, and *A. fumigatus*. [DipR]_5_ showed promising MIC values of 0.39–6.3 µM (0.74–11.9 µg/mL) against Gram-positive bacteria strains, namely *MRSA*, *S. aureus*, *E. faecium*, *E. faecalis*, *S. pneumoniae*, and *B. subtilis* bacteria. On the other hand, [DipR]_5_ showed modest MIC values of 12.5–25 µM (23.78–47.56 µg/mL) against Gram-negative strains, namely *K. pneumoniae*, *P. aeruginosa*, and *E. coli*. For antifungal studies, [DipR]_5_ showed promising MIC values of 1.6–6.6 µM (3–12.5 µg/mL) against *C. parapsilosis* and *A. fumigatus* and was significantly more potent than fluconazole against the latter. These data provide insights into designing novel antibacterial and antifungal peptides containing arginine, tryptophan, and diphenylalanine. A deeper analysis of the structural differences between these peptides, such as the arrangement of tryptophan, arginine, and other residues, could provide insights into the critical structural features contributing to their antibacterial and antifungal properties. Further optimization of these compounds and in vivo studies will be crucial to fully assess their therapeutic potential as broad-spectrum antimicrobial agents. Future studies could include complementary assays to provide a more comprehensive understanding of the antimicrobial activity of these peptides.

## Figures and Tables

**Table 1:** Minimum inhibitory concentration (µM) of ten linear and cyclic peptides against Gram-positive and Gram-negative bacteria.

	MRSA (ATCC BAA-1556)	*S. aureus* (ATCC 29213)	*E. faecium* (ATCC 700221)	*S. pneumoniae* (ATCC 51938)	*E. faecalis* (ATCC 29212)	*B. subtilis* (ATCC-6633)	*K. pneumoniae* (ATCC BAA-1705)	*P. aeruginosa* (ATCC 27883)	*E. coli* (ATCC 25922)
MIC (µM) ^a^									
**((DipR)_4_(WR))**	3.1	3.1	1.6	0.78	1.6	12.5	25	25	12.5
**((DipR)_3_(WR)_2_)**	3.1	3.1	3.1	0.78	3.1	3.1	25	25	12.5
**((DipR)_2_(WR)_3_)**	3.1	3.1	3.1	0.78	6.3	1.6	12.5	12.5	6.3
**((DipR)(WR)_4_)**	6.3	6.3	3.1	0.78	12.5	3.1	12.5	25	12.5
**(DipR)_4_R**	1.6	1.6	1.6	0.39	3.1	1.6	6.3	12.5	6.3
**[(DipR)_4_(WR)]**	3.1	NT ^b^	NT	NT	NT	NT	25	25	12.5
**[(DipR)_3_(WR)_2_]**	3.1	NT	NT	NT	NT	NT	12.5	25	12.5
**[(DipR)_2_(WR)_3_]**	3.1	NT	NT	NT	NT	NT	12.5	25	12.5
**[(DipR)(WR)_4_]**	3.1	NT	NT	NT	NT	NT	12.5	25	12.5
**[DipR]_5_**	3.1	3.1	0.78	0.39	0.78	6.3	25	25	12.5
**Daptomycin**	1.2	0.62	1.2	4.9	9.87	0.3	NA ^c^	NA	NA
**Meropenem**	5.2	0.5	83.0	0.6	5.2	0.6	42	2.6	2.6

^a^ All experiments were conducted in triplicate and repeated twice; ^b^ NT = Not tested; ^c^ NA = Not active.

**Table 2:** Minimum inhibitory concentration (µM) of ten linear and cyclic peptides against *A. fumigatus*, *C. albicans*, and *C. parapsilosis*.

	*C. albicans* (ATCC 60193)	*C. parapsilosis* (ATCC 22019)	*A. fumigatus* (Af-293)
	MIC (µM) ^a^		
**((DipR)_4_(WR))**	6.6	6.6	1.6
**((DipR)_3_(WR)_2_)**	3.4	6.8	1.7
**((DipR)_2_(WR)_3_)**	3.5	6.9	3.5
**((DipR)(WR)_4_)**	28.2	14.1	3.5
**(DipR)_4_R**	7.4	7.4	1.8
**[(DipR)_4_(WR)]**	6.7	6.7	1.7
**[(DipR)_3_(WR)_2_]**	6.8	6.8	1.7
**[(DipR)_2_(WR)_3_]**	7.0	7.0	1.7
**[(DipR)(WR)_4_]**	14.3	7.1	1.8
**[DipR]_5_**	13.1	6.6	1.6
**Fluconazole**	4.9	3.3	>209
**Amphotericin B**	0.42	0.42	0.84

^a^ All experiments were conducted in triplicates and repeated twice.

## Data Availability

The original contributions presented in this study are included in the article. Further inquiries can be directed to the corresponding author.
